# Theoretical study of the adsorption characteristics and the environmental influence of ornidazole on the surface of photocatalyst TiO_2_

**DOI:** 10.1038/s41598-019-47379-y

**Published:** 2019-07-26

**Authors:** Ruolan Tan, Zhongjian Lv, Jing Tang, Yiwei Wang, Jianmin Guo, Laicai Li

**Affiliations:** 1 College of Pharmacy, Southwestern Medical University, Luzhou, 646000 China; 2 Chengdu Clementine Pharmaceutical Technology Co., Ltd, Chengdu, 610000 China; 3 College of Basic Medical Sciences, Southwestern Medical University, Luzhou, 646000 China; 4 College of Chemistry and Material Science, Sichuan Normal University, Chengdu, 610066 China

**Keywords:** Density functional theory, Quantum chemistry

## Abstract

In this paper, density functional theory (DFT) was performed to study the adsorption properties of ornidazole on anatase TiO_2_(101) and (001) crystal facets under vacuum, neutral and acid-base conditions. We calculated the adsorption structure of ornidaozle on the anatase TiO_2_ surface, optimal adsorption sites, adsorption energy, density of states, electronic density and Milliken atomic charge under different conditions. The results show that when the N(3) atom on the imidazole ring is adsorbed on the Ti(5) atom, the largest adsorption energy and the most stable adsorption configuration could be achieved. According to the analysis of the adsorption configuration, we found that the stability of C(2)-N(3) bond showed a weakening trend. The adsorption wavelengths of the electronic transition between the valence band and conduction band of ornidazole on the TiO_2_ surface were in the visible light wavelengths range, showing that the TiO_2_ crystal plane can effectively make use of visible light under different conditions. We speculate the possibility of ornidazole degradation on the surface of TiO_2_ and found that the reactive site is the C-N bond on the imidazole ring. These discoveries explain the photocatalytic degradation of ornidazole by TiO_2_ and reveal the microscopic nature of catalytic degradation.

## Introduction

Ornidazole (1-(3chloro-2-hydroxypropyl)-2-methyl-5-nitroimidazole) is a third-generation nitroimidazole drug with anti-anaerobic activity that is commonly used to treat trichomoniasis and amoeba infections^1^. For pharmaceuticals taken by humans and animals, most of the dose is excreted in the form of urine and feces as the original drug or metabolites^2^. Due to the high water-solubility and low biodegradation rates of residual drugs, they are not easily degraded in the environment and are ultimately enriched in water body^3^. The remaining antibiotics in the water environment can be accumulated in human bodies through the food chain even at low concentrations^4^. Drug toxicology experiments have shown that these drugs have potential hazards (genotoxicity^5^, neurotoxicity^6^, mutagenic^7^, etc.). The residues also produce resistant bacteria to interfere with ecosystem stability^8^. Therefore, how to remove antibiotic residues in the environment is of great importance. Although many methods for the degradation of targeted drugs are reported in the literature (absorption, biodegradation, and chemical oxidation), low concentrations of residual drugs are difficult to remove and may cause secondary pollution. Therefore, the use of these methods is subjected to restrictions.

TiO_2_ photocatalysis is an advanced oxidation technology using strong oxidizing species such as hydroxyl radicals (·OH), superoxide anions (·O_2_) and perhydroxyl radicals (HOO·) to mineralize organic matter into carbon dioxide, water and inorganic ions^9,10^. At the same time, TiO_2_ photocatalysis has been applied in many ways, such as the degradation of industrial dyes, pesticide residues, drug residues, etc., due to its mild reaction conditions, no secondary pollution and no toxic byproduct properties. Mohammad *et al*.^11^ adopted anatase TiO_2_ as a photocatalyst to degrade two types of reactive azo dyes. By continuously changing the test parameters, the two dyes could be completely degraded. Combining quantum chemical theory calculations with experiments, Liu^12^ employed the yttrium-doped TiO_2_ (TiO_2_/Ce) hydrosol as a photocatalyst to analyze the degradation effect of the pesticide residue dimethoate, and conducted meritorious studies on the subsequent pesticide residues as well. Marothu^13^ found that the heterogeneous photocatalytic degradation technology is very effective towards the anti-Parkinson–like entacapone with anatase TiO_2_, and they studied the effects of the parameters of degradation, such as the catalyst loading, acidity and alkalinity of the solution, and initial concentration. In the present study, anatase TiO_2_ was utilized as a catalyst to study the adsorption properties of ornidazole on the TiO_2_(101) and (001) facets under different conditions. We hope to supply some theoretical information for the research of ornidazole.

## Results and Discussion

The molecular structure of ornidazole (seen in Fig. S1) and the stable crystal planes of TiO_2_(101) and (001) were optimized. The molecular dynamics of ornidazole on the TiO_2_(101) and (001) crystal facets was simulated by the LAMMPS program. Based on the relaxation results, we selected the relatively stable adsorption configurations to further optimize the molecular structures by the Materials Studio program. The adsorption energies for the adsorption configurations are shown in Table 1.

**Table 1:** Adsorption energies for the adsorption configurations of ornidazole adsorbed on TiO_2_(101) and (001) facets.

Condition	(101) surface		(001) surface		Condition	(101) surface		(001) surface	
	Compound	*E*_ads_	Compound	*E*_ads_		Compound	*E*_ads_	Compound	*E*_ads_
Vacuum conditions	A1	1.30	a1	2.68	Acid conditions	C1	3.04	c1	2.81
	A2	0.91	a2	2.18		C2	1.95	c2	2.88
	A3	0.89	a3	2.63		C3	2.10	c3	2.60
	A4	0.86	a4	1.91		C4	2.13	c4	2.41
	A5	1.16	a5	2.55		C5	2.21	c5	2.51
Neutral conditions	B1	2.45	b1	2.64	Basic conditions	D1	2.35	d1	2.42
	B2	2.00	b2	2.41		D2	2.03	d2	2.56
	B3	2.19	b3	2.39		D3	1.89	d3	1.72
	B4	2.45	b4	2.45		D4	2.45	d4	2.64
	B5	2.59	b5	2.52		D5	2.52	d5	2.89

### Adsorption under vacuum conditions

**Figure 1:**
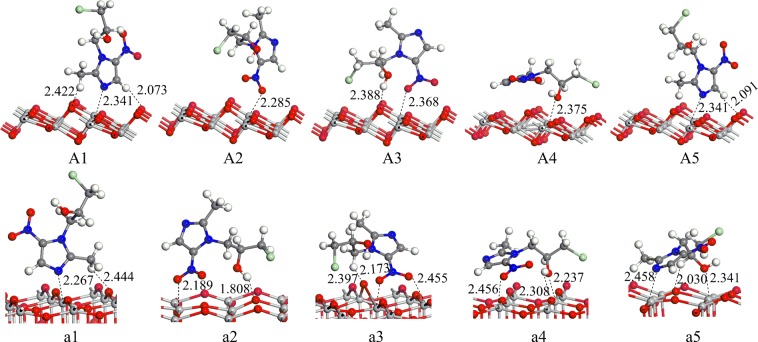
The adsorption structures of ornidazole on the TiO_2_(101) and (001) facets under vacuum conditions.

As shown in Fig. 1, A1~A5 and a1~a5 are five stable configurations of ornidazole adsorbed on TiO_2_(101) and (001) facets under vacuum conditions, respectively. The nitro moiety O atom and the hydroxyl group O atom on the imidazole ring can absorb on the Ti(5) atom. The H atoms of the C(2) methyl group, on the N(1) branch and on C(4) can form hydrogen bonds with the O(2) atom. Such bonds do not exist that for the N(3) atom adsorbed on the Ti(6) atom and the H atom adsorbed on the O(3) atom. Thus, it is shown that the Ti(5) and O(2) atoms are more active than the Ti(6) and O(3) atoms. Some bond lengths on the surface of TiO_2_ are slightly deformed owing to the interaction of the ornidazole molecule with the TiO_2_ surface. Zhang^14^ founded that the hydrogen bond can enhance the stability of the multilayer dye aggregates on the TiO_2_ surface. The study by Chang showed that the hydrogen bond between HNO_3_ and TiO_2_ can enhance the adsorption energy and the stability of the adsorption configuration^15^. It is observed that the adsorption configuration can be stabilized by the formation of hydrogen bonds.

From Table 1, can be observed that under the vacuum conditions, A1 is the most stable adsorption configuration on the TiO_2_(101) surface. For A1 mode, the N(3) atom adsorbed on the Ti(5) atom, and the H atom of the methyl moiety and the C(2) atomic branch form hydrogen bonds with the O(2) atom on the TiO_2_(101) plane. The adsorption distances are 2.341, 2.073 and 2.422 Å, respectively. Due to the interaction of the ornidazole molecule with the surface of TiO_2_, some bond lengths are changed. C(2)-N(3) and N(3)-C(4) in the imidazole ring change from 1.335 and 1.354 Å to 1.352 and 1.361 Å, respectively, and the C(2)-N(3)-C(4) bond angle changes from 106.1°to 107.3°. The bond length of C(2)-N(3) increases even more than the N(3)-C(4), which means that the process of adsorption make C(2)-N(3) more unstable and favors the attack of the hydroxyl radicals.

Similarly, Table 1 shows that under vacuum conditions, a1 is the most favorable configuration with the highest adsorption energy about 2.83 eV. In a1 configuration (Fig. 1), the N(3) atom adsorbed on the Ti(5) atom and the H atom on the C(2) atomic branch of ornidazole adsorbed on the O(2) atom. The adsorption distances are 2.267 and 2.444 Å, respectively. The C(2)-N(3) bond length in the imidazole ring has been greatly changed due to the adsorption, increasing from 1.335 Å to 1.346 Å. This result indicates that the stability of C(2)-N(3) bond weakens, which favors the attack of the hydroxyl radicals and ring opening degradation.

### Adsorption under solvent conditions

To take into account the adsorption characteristics of the ornidazole molecule on the TiO_2_ crystal surface under solvent conditions, we used the same method to optimize the stable adsorption structures of ornidazole on the TiO_2_(101) and (001) crystal facets under solvent conditions.

### Adsorption under neutral conditions

**Figure 2:**
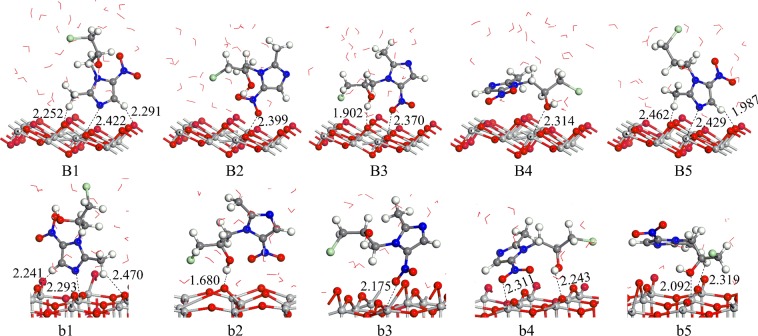
The adsorption structures of ornidazole on the TiO_2_(101) and (001) facets in neutral conditions. The H_2_O molecules are represented by bond line notation.

The modes of B1~B5 and b1~b5 are shown in Fig. 2, under neutral solvent conditions. The adsorption of ornidazole on the TiO_2_ surface is still multisite adsorption. Due to the addition of the water solvent model, the H atoms of H_2_O molecules form hydrogen bonds with O atoms on the crystal surface, and the O atoms of H_2_O molecules and Ti atoms form Ti-O and Ti-OH bonds on TiO_2_(101) and (001) surfaces, respectively. The degree of deformation of the crystal facets is greater than that under vacuum conditions to maintain the stability of the adsorption configuration. In terms of the adsorption energies (Table 1), the B5 and b1 configurations are the most stable structures of ornidazole on the anatase TiO_2_(101) and (001) crystal facets, which are 2.45 and 2.64 eV, respectively. The adsorption characteristics of B5 and b1 are similar to those of the A1 and a1 configurations, and the stability of C(2)-N(3) bond tends to be weak and is susceptible to attack by hydroxyl radicals. After adsorption the N(3)-C(2) bond length become longer compare to vacuum conditions. In the solvent conditions, H_2_O molecules are revolved around ornidazole, there may be strong interactions between H_2_O molecules and the ornidazole molecule. Meanwhile, the adsorption energy increase relative to the vacuum conditions. Zhang^16^ *et al*. studied the adsorption of CO atoms on the CuCl(111) surface in solvent condition. Mendive^17^ studied the adsorption of oxalate on anatase(100) and rutile(110) in aqueous solution. The water solvent stabilized the adsorption structure and also illustrates the effect of water solvent on the adsorption energy of the surface.

### Adsorption under acidic conditions

**Figure 3:**
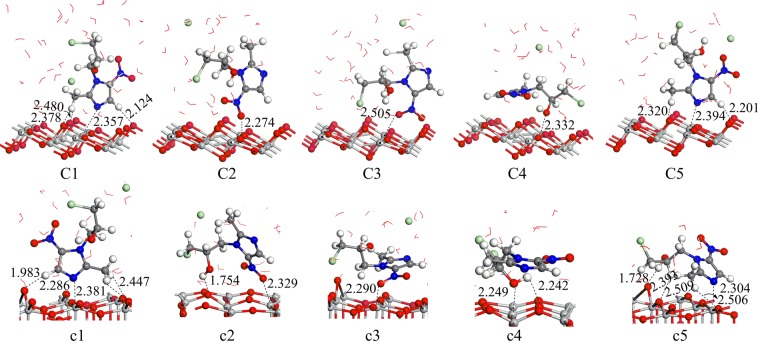
The adsorption modes of ornidazole on the TiO_2_(101) and (001) facets in acid conditions. The H_2_O molecules are represented by bond line notation, and the Cl atom is represented by green circles.

Figure 3 shows the adsorption distances and sites of C1~C5 and c1~c5 configurations under acidic conditions. Due to the interactions of the ornidazole molecule, water molecules, proton and chloride ion with TiO_2_, a subtle deformation of the TiO_2_ crystal plane occurs. As shown in Table 1, in terms of the adsorption energy, C1 mode is the most stable configuration under acidic conditions. The adsorption properties of C1 are similar to those of A1 except that more hydrogen bonds have formed. The c2 configuration has the largest adsorption energy, 2.89 eV, and is the most stable adsorption configuration of ornidazole on the TiO_2_(001) surface under acidic conditions. In the c2 configuration, the N(3) atom of ornidazole is not adsorbed on the Ti(5) atom. However, by investigating the bond lengths of the c2 configuration, we found that the bond lengths of C(2)-N(3) and C(4)-C(5) increase after adsorption from 1.335 and 1.384 Å to 1.354 and 1.398 Å, respectively. The length of C(2)-N(3) is longer than that of C(4)-C(5). At the same time, we also analyzed the other four configurations and found that the bond lengths of the ornidazole molecule change differently and that the C(2)-N(3) bond length obviously increases, disclosing adsorption makes the stability of C(2)-N(3) weaker and more susceptible to attack by hydroxyl radicals.

### Adsorption under alkaline conditions

**Figure 4:**
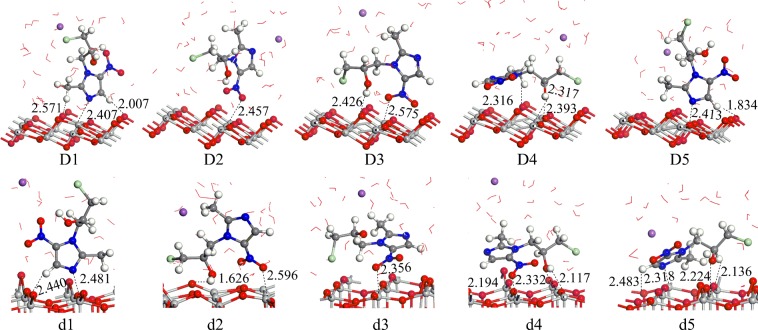
The adsorption modes of ornidazole on the TiO_2_(101) and (001) facets in basic solution. The H_2_O molecules are represented by bond line notation, and the Na atom is represented by purple circles.

As demonstrated in Fig. 4, D1~D5 and d1~d5 are five adsorption configurations of ornidazole on the TiO_2_(101) and (001) facets under alkaline conditions, respectively. From Table 1 we observe that D5 (adsorption energy of 2.52 eV) is the most stable adsorption configuration of ornidazole on the TiO_2_(101) surface. Similarly, in terms of the adsorption energy (shown in Table 1), d5 (2.89 eV) is the most stable adsorption configuration of ornidazole on the TiO_2_(001) surface. The adsorption properties of the D5 and d5 configurations are also similar to those of A1 and a1 under vacuum conditions, except that in the d5 structure, more hydrogen bonds form and the O atom in the hydroxyl moiety interacts with Ti(5). Adsorption also makes the stability of C(2)-(N3) weaker and thus susceptible to attack by hydroxyl radicals and ring-opening degradation.

By analyzing the adsorption energy and adsorption configuration of the ornidazole molecule on the TiO_2_ crystal surface under vacuum and aqueous conditions, we found that the adsorption configuration is more stable under aqueous conditions. The adsorption of ornidazole on the TiO_2_ surface is affected by the intermolecular surface tension and hydrogen bonding. After adsorption, the crystal surface is slightly deformed due to the influence of the ornidazole molecule, water molecules, proton and ion on the crystal plane of TiO_2_. We also found that when the ornidazole molecule adsorbed on the TiO_2_(001) crystal plane, the degree of deformation of the crystal plane is much greater than that of the (101) plane under vacuum or aqueous solution conditions, which may be related to the fact that the anatase TiO_2_(001) crystal plane has more unsaturated titanium ions and a higher surface activity^18–20^. Therefore, the (001) surface may be more favorable for photocatalysis. Additionally, the overall adsorption energy of ornidazole is found to be the highest under acidic conditions. The isoelectric point of TiO_2_ is 6.3^21^, which indicates that the positive charge on the surface of TiO_2_ is beneficial to the adsorption of the ornidazole molecule when the pH is less than 6.3. In contrast, the negative charge on the surface of TiO_2_ is not conducive to the adsorption of the ornidazole molecule. The results may provide a certain reference for the degradation conditions of ornidazole.

On the basis of the characteristics of the molecular adsorption structure, we found that the most stable adsorption configuration of ornidazole on two surfaces of TiO_2_ is the N(3) atom adsorbed on the Ti(5) atom. After the ornidazole adsorbed on TiO_2_ surface, the bond length of C-N much longer. Thus, according to the adsorption results, it is reasonable to speculate that the ring-opening reaction site of ornidazole is the C-N bond^22–24^.

### Electronic structure

To further investigate the interaction and bond characteristics of the ornidazole molecule with the TiO_2_ crystal plane, we calculated the density of states (DOS), projected density of states (PDOS), electron density, and Milliken atomic charge of adsorption configurations under vacuum and aqueous conditions. The DOS and PDOS of the TiO_2_(101) and (001) facets consist of the 2p and 3d valence bands (VB) of O and Ti, while the conduction bands (CB) are primarily composed of the 3d orbital of Ti.

The DOS and PDOS of the ornidazole-adsorbed TiO_2_ surface under vacuum conditions are giving in Fig. S2 and Fig. S3. From these figures we found that the s-orbital composition of the TiO_2_(001) plane is even more than that of the TiO_2_(101) plane. For semiconductor photocatalytic materials, the electronic transition between the CB and VB is affected by the energy gap. If the energy gap is in the visible light range, visible light can be effectively utilized. Therefore, the energy gaps of different crystal plane adsorption configurations, which are the differences between the highest occupied molecular orbital (HOMO) and lowest unoccupied molecular orbital (LUMO) energies, can be used to judge the utilization of visible light. By calculating the energy gap, their values are the difference. The calculated energy gap of bulk TiO_2_ is 2.87 eV, which is close to the experimental value of 3.20 eV^25^. After adsorption, the energy gap became narrower. The energy gap values of A1~A5 are 2.03, 2.27, 1.94, 1.96, and 2.00 eV, respectively. In the structures of a1~a5, the energy gap values are 1.98, 1.61, 1.75, 1.56, and 2.03 eV, respectively. The electronic transition wavelength between the VB and the CB corresponds to visible light (the visible photon energy gap range is approximately 1.7~3.1 eV)^26,27^, demonstrating that using visible light to drive the degradation of ornidazole on TiO_2_ surface is effective.

The DOS and PDOS of adsorption configuration under neutral aqueous conditions are plotted in Figs S4 and S5. The band structure of TiO_2_ changes due to the action of H_2_O molecules on TiO_2_ surface, in the water solvent conditions. After adsorption, the Ti 3d states still govern the CB edge, and the s-orbital component of the VB energy level is increased. Two peaks of s orbital form between −21 eV and −15 eV, and the energy range of the p orbital is broadened from −5~0 eV to −8~0 eV. From −8~0 eV, two sets of peaks appear, in which the peak height and peak area are increased compared to that of pure TiO_2_. The increase in the s-orbital and p-orbital components elucidates that the 1 s orbital of the H atom and the 2p orbital of the O atom in the H_2_O molecules participate in hybridization. The s orbital appears near the Fermi level, which corresponds to the HOMO orbital of the system. When the number of electrons in the HOMO or LUMO orbital increase, the electron donating ability of the system also increase, showing that the chemical activity of TiO_2_ is improved. As well, the TiO_2_(001) surface has more p-orbital components, indicating that there may be more 2p orbitals of O in the H_2_O molecules involving in the hybridization and that the chemical activity of the TiO_2_(001) surface is greater than that of the (001) plane. The TiO_2_ energy gap is narrowed after adsorption. The energy gaps of B1~B5 are reduced to 2.313, 2.153, 2.121, 2.331, and 2.251 eV. The b1~b5 energy gaps are reduced to 2.127, 1.704, 2.005, 2.029, and 2.077 eV, respectively. The above energy gaps all in the visible light range, showing that the TiO_2_ surface can effectively utilize visible light under water solvent conditions.

The DOS and PDOS for the adsorption of ornidazole on anatase TiO_2_(001) and (001) facets under acidic conditions are shown in Figs S6 and S7, respectively. Clearly, the TiO_2_ band structure is similar to that of the neutral solution. The energy gap is narrower after adsorption than that of pure anatase TiO_2_. The C1~C5 energy gaps are reduced to 2.301, 2.306, 1.900, 2.052, and 1.162 eV, respectively. The light absorption frequency is reduced, except for the C5 configuration, and they are all in the visible wavelength range. The energy gaps of c1~c5 are 1.789, 1.824, 1.722, 2.131, and 1.958 eV, respectively, which are in the visible light range. These results show that, under acidic conditions, the TiO_2_ crystal surface can effectively use visible light.

The DOS and PDOS of adsorbed-TiO_2_ under alkaline conditions are shown in Figs S8 and S9, respectively. The band structure is similar to that of the neutral conditions. Adsorption results in the narrowing of the TiO_2_ energy gap. Specifically, the D1~D5 energy gaps are reduced to 2.325, 1.978, 1.640, 2.327, and 2.336 eV, respectively, and the d1~d5 energy gaps are 1.990, 2.043, 1.700, 1.932 and 1.930 eV, respectively. All of the energy gaps are in the visible range, and thus, the TiO_2_ crystal plane can effectively use visible light, under basic conditions.

**Figure 5:**
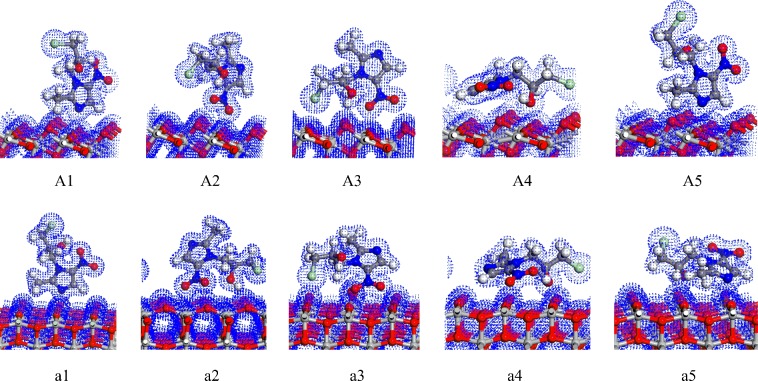
Electronic configurations of ornidazole on TiO_2_(101) and (001) facets.

Figure 5 shows the electron density of ornidazole on the TiO_2_ crystal surface under vacuum conditions. We observed an overlap between the charge density of the imidazole ring and the TiO_2_ surface. From Table S1, we can see that under different conditions, the number of electrons on the imidazole ring increases after adsorption, which shows that the electrons of the crystal surface have shifted to the imidazole ring during the adsorption process. These results demonstrate that electron transfer occurs between the imidazole ring and TiO_2_ surface and that a new chemical bond is formed. During the process of adsorption, the ornidazole molecule interacts with the surface of TiO_2_ and undergoes chemical adsorption.

## Conclusion

In this work, DFT was used to study the adsorption characteristics of ornidazole on the anatase TiO_2_(101) and the (001) facets under different conditions. The result showed that ornidazole can absorb on the TiO_2_ surface in vacuum or aqueous solution conditions, especially acidic conditions. After adsorption the bond length of C-N in the imidazole ring becomes longer, which is conducive to the attack and ring-opening degradation of the hydroxyl radicals. Through the molecular adsorption structure change characteristics, we found that the reaction site of degradation is the ring-opening of the C-N bond on the imidazole ring. At the same time, the hydrogen bonding played a role in the process of ornidazole adsorbed on the surface of TiO_2_. Compared with vacuum conditions, the hydrogen bonding effect in the adsorption process under aqueous conditions is more significant for the change in the adsorption characteristics. For different conditions, we found that the adsorption wavelengths of the electronic transition between the VB and CB of each adsorption configuration on the TiO_2_(101) and (001) crystal facets correspond to visible light. Our results reveal that the TiO_2_ can effectively use visible light and can be used as a photodegradation catalyst for ornidazole.

## Methods

**Figure 6:**
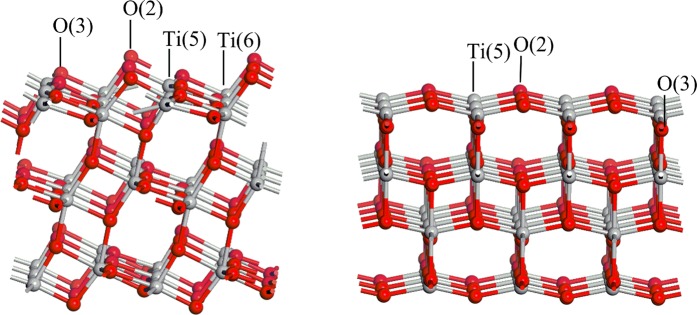
The model of anatase TiO_2_(101) and (001) crystal facets.

The anatase TiO_2_(101) and (001) crystal facets were investigated in this paper. From Fig. 6, it is can be observed that the surface of TiO_2_ show 5-fold and 6-fold coordinated Ti atoms (Ti(5) and Ti(6)), as well as 2-fold and 3-fold coordinated oxygen atoms (O(2) and O(3)). Notably, Ti(6) site does not exist in TiO_2_(001) surface layer. Based on a preliminary study of the effect of the plate thickness on the surface energy, when the (101) surface adopts a three-layer model^28^ and the (001) surface adopts a layer model^29,30^, the calculation time and accuracy can be balanced. To avoid the interaction between the molecule and the plate, a 15 Å vacuum layer in the Z direction was added. The (1 × 3) supecell and (3 × 3) supecell were used for antase TiO_2_(101) and (001) surfaces with a (TiO_2_)_36_ composition. The corresponding surface areas are 10.886 Å × 11.328 Å and 11.328 Å × 11.328 Å on the (101) and (001) surfaces of TiO_2_, respectively. In neutral aqueous solution, under the Universal force field and according to the density of 1 g/cm^3^, 48 and 69 H_2_O molecules are approximately added on the surfaces of TiO_2_(101) and (001), respectively. In acidic (basic) conditions, one H_2_O molecule was replaced by with a molecule of HCl (NaOH)^31^.

Placing the ornidazole molecule on the TiO_2_(101) and (001) crystal facets, the distance between them was set to approximately 3.8 Å to avoid a strong interaction, and then we introduced the reactive force field (ReaxFF) and NVE ensemble under the LAMMPS program to perform a molecular dynamic calculation^32,33^. Based on the LAMMPS relaxation results, the local minimum structure was selected for further optimization by DFT.

The DFT calculation was performed using the DMol3 code of the MS package^34^. DMol3 applied the dual digital base group and polarization function to extend the electronic wave function and all structural optimization was performed on the basis of spin-polarized plane waves. The Kohn-Sham one-electron equations were solved in the generalized gradient approximation (GGA) by using the Perdew-Burke-Ernzerhof (PBE) functional^35^, and the effective core potentials (ECP) was used to describe the core electrons. The polarized DNP^36^ basis set was used to describe the atomic orbitals and the cutoff radius was set to 4.5 Å. For the calculation of the adsorption results, the convergence criterion is set to the following criteria: the energy was smaller than 2 × 10^−5^ Ha, the force was below 4 × 10^−3^ Ha/Å, and the max displacement was 5 × 10^−3^ Å. In addition, the self-consistent field (SCF) iterative energy tolerance was set to 1 × 10^−5^ Ha, and the multipole expansion was performed by the octupole moment.

The adsorption energy (*E*_*ads*_) is defined as

$${E}_{ads}=({E}_{surf}+{E}_{mol})-{E}_{total}$$

where *E*_*total*_ is the free energy for the ornidazole molecule absorbed on TiO_2_ surface, *E*_*surf*_ is the energy of the TiO_2_ surface, and *E*_*mol*_ is the energy for the ornidazole molecule.

The lattice parameters of bulk anatase TiO_2_ optimized by the above method are a = b = 3.776 Å, and c = 9.486 Å, which are consistent with the experimental values of a = b = 3.785 Å and c = 9.514 Å^37,38^. The agreement shows that our calculation method and results are reliable.

## Supplementary information

Supplementary Dataset 1

## Data Availability

Data related to the article can be obtained from the author.

## References

[1] Chandrasekarana K, Thilak KR (2016). Molecular properties prediction, docking studies and antimicrobial screening of ornidazole and its derivatives. J. Chem. Pharm. Res..

[2] Kern JK (2013). Thimerosal Exposure and the Role of Sulfation Chemistry and Thiol Availability in. Autism. Int. J. Environ. Res. Public. Health..

[3] Karunaratne DN (2017). Nanotechnological Strategies to Improve Water Solubility of Commercially Available Drugs. Curr. Nanomed..

[4] Martínez C, Canle ML, Fernández MI, Santaballa JA, Faria J (2011). Kinetics and mechanism of aqueous degradation of carbamazepine by heterogeneous photocatalysis using nanocrystalline TiO_2_, ZnO and multi-walled carbon nanotubes–anatase composites. Appl. Catal. B-Environ..

[5] Ikbal M, Yilmaz G, Dogan H, Alp MY, Cebi AH (2011). The evaluation of genotoxic potential of ornidazole, nitroimidazole, in lymphocyte culture of patients with amebiasis. Drug. Chem. Toxicol..

[6] De MM (2010). Evaluation of the mutagenic and genotoxic activities of 48 nitroimidazoles and related imidazole derivatives by the Ames test and the SOS chromotest. Environ. Mol. Mutagen..

[7] Ferreiroa GR (2002). DNA single strand breaks in peripheral blood lymphocytes induced by three nitroimidazole derivatives. Toxicol. Lett..

[8] Port JA, Cullen AC, Wallace JC, Smith MN, Faustman EM (2014). Metagenomic frameworks for monitoring antibiotic resistance in aquatic environments. Environ. Health. Perspect..

[9] Sleiman M, Conchon P, Ferronato C, Chovelon JM (2007). Iodosulfuron degradation by TiO_2_ photocatalysis: kinetic and reactional pathway investigations. Appl. Catal. B-Environ..

[10] Xia T, Zhang YL, Murowchickc J, Chen XB (2014). Synthesis and photoactivity of nanostructured CdS–TiO_2_, composite catalysts. Catal. Today..

[11] Mohammad SM, Ashaduzzaman M, Rashid TU, Dey SC, Amin MA (2016). Solar Assisted Photocatalytic Degradation of Reactive Azo Dyes in Presence of Anatase Titanium Dioxide. Int. J. Lat. Res. Eng. Techno..

[12] Liu Xiangying, Li Yu, Zhou Xuguo, Luo Kun, Hu Lifeng, Liu Kailin, Bai Lianyang (2018). Photocatalytic degradation of dimethoate in Bok choy using cerium-doped nano titanium dioxide. PLOS ONE.

[13] Marothu VK, Nellutla A, Gorrepati M, Majetib S, Mamidalabet SK (2015). Forced degradation studies, and effect of surfactants and titanium dioxide on the photostability of paliperidone by HPLC. Ann. Pharm. Fr..

[14] Zhang L, Liu XG, Rao WF, Li JF (2016). Multilayer Dye Aggregation at Dye/TiO_2_ Interface via π…π Stacking and Hydrogen Bond and Its Impact on Solar Cell Performance: A DFT Analysis. Sci. Rep..

[15] Chang CY, Chen HT, Lin MC (2009). Adsorption Configurations and Reactions of Nitric Acid on TiO_2_ Rutile (110) and Anatase (101) surfaces. J. Phys. Chem. C..

[16] Zhang RG, Ling LX, Wang BJ, Huang W (2010). Solvent effects on adsorption of CO over CuCl(111) surface: A density functional theory study. Appl. Surf. Sci..

[17] Mendive CB, Bredow T, Feldhoff A, Blesa MA, Bahnemann D (2009). Adsorption of oxalate on anatase (100) and rutile (110) surfaces in aqueous systems: experimental results vs. theoretical predictions. Phys. Chem. Chem. Phys..

[18] Liu SW, Yu JG, Jaroniec M (2010). Tunable Photocatalytic Selectivity of Hollow TiO_2_ Microspheres Composed of Anatase Polyhedra with Exposed {001} Facets. J. Am. Chem. Soc..

[19] Gao BF (2014). Facile Synthesis of TiO_2_ Microspheres with Reactive (001) Facets for Improved Photocatalytic Performance. J. Nanosci. Nanotechno..

[20] Chen JS (2010). Constructing hierarchical spheres from large ultrathin anatase TiO_2_ nanosheets with nearly 100% exposed (001) facets for fast reversible lithium storage. J. Am. Chem. Soc..

[21] Chen SL, Diane L, Mark S (2001). Enhancement of the electrochemical oxidation of formic acid. Effects of anion adsorption and variation of rotation rate. Electrochim. Acta..

[22] Acosta-Rangel A, Sánchez-Polo M, Polo AMS, Rivera-Utrilla J, Berber-Mendoza MS (2018). Tinidazole degradation assisted by solar radiation and iron-doped silica xerogels. Chem. Eng. J..

[23] Zhao J, Yao BH, He Q, Zhang T (2012). Preparation and properties of visible light responsive Y^3+^ doped Bi_5_Nb_3_O_15_ photocatalysts for Ornidazole decomposition. J. Hazard. Mater..

[24] Wang DY, Luo H, Liu LX, Wei W, Li LC (2019). Adsorption characteristics and degradation mechanism of metronidazole on the surface of photocatalyst TiO_2_: A theoretical study. Appl. Surf. Sci..

[25] Nasr M (2017). Enhanced Visible-Light Photocatalytic Performance of Electrospun rGO/TiO_2_ Composite Nanofibers. J. Phys. Chem. C..

[26] Yaghoubi H (2015). Toward a Visible Light-Driven Photocatalyst: The Effect of Midgap-States-Induced Energy Gap of Undoped TiO_2_ Nanoparticles. Acs. Catal..

[27] Kurian S, Seo H, Jeon H (2013). Significant Enhancement in Visible Light Absorption of TiO_2_ Nanotube Arrays by Surface Band Gap Tuning. J. Phys. Chem..

[28] O’Rourke C, Bowler DR (2010). Adsorption of Thiophene-Conjugated Sensitizers on TiO_2_ Anatase (101). J. Phys. Chem. C..

[29] Vittadini A, Selloni A, Rotzinger FP, Grätzel M (1998). Structure and Energetics of Water Adsorbed at TiO_2_ Anatase (101) and (001) Surfaces. Phys. rev. lett..

[30] Ma JG (2014). The adsorption of α-cyanoacrylic acid on anatase TiO_2_ (101) and (001) surfaces: A density functional theory study. J. Chem.Phys..

[31] Agosta L, Brandt EG, Lyubartsev AP (2017). Diffusion and reaction pathways of water near fully hydrated TiO_2_ surfaces from ab initio molecular dynamics. J. Chem. Phys..

[32] Rahaman O, Duin ACTV, Goddard WA, Doren DJ (2011). Development of a ReaxFF reactive force field for glycine and application to solvent effect and tautomerization. J. Phys. Chem. B..

[33] Plimpton S (1995). Fast parallel algorithms for short-range molecular dynamics. J. Comput. Phys..

[34] Day GM, Motherwell WDS, Jones W (2007). A strategy for predicting the crystal structures of flexible molecules: the polymorphism of Phenobarbital. Phys. Chem. Chem. Phys..

[35] Ichiya T (2010). Surface Electronic/Atomic Structure and Activation Energy on Pt(111), Pt_3_Cu(111), and PtCu(111) for PEFC Cathode. Nanosc. Microsc. Therm..

[36] Kresse G, Furthmüller J (1996). Efficiency of ab-initio total energy calculations for metals and semiconductors using a plane-wave basis set. Comput. Mater. Sci..

[37] Abrahams SC, Bernstein JL (1969). Remeasurement of the structure of hexagonal ZnO. Acta. Cryst..

[38] Cromer DT, Herrington K (1955). The Structures of Anatase and Rutile. J. Am. Chem. Soc..

