# Supplementary material for: Theoretical study of the adsorption characteristics and the environmental influence of ornidazole on the surface of photocatalyst TiO2
Source: Sci Rep. 2019 Jul 26;9:10891. doi: 10.1038/s41598-019-47379-y (PMC6659643; doi:10.1038/s41598-019-47379-y)
Supplement: Supplementary file 1 — Supplementary Dataset 1 [file 41598_2019_47379_MOESM1_ESM.doc]

**Theoretical study of the adsorption characteristics and the environmental influence of ornidazole on the surface of photocatalyst TiO_2_**

Ruolan Tan^1^, Zhongjian Lv^2^, Jing Tang^1^, Yiwei Wang^3^, Jianmin Guo^3,*^ & Laicai Li^4,*^

(^1^ College of Pharmacy, Southwestern Medical University, Luzhou 646000, China)

(^2^ Chengdu Clementine Pharmaceutical Technology Co., Ltd ,Chengdu 610000, China)

(^3^ *College of Basic Medical Sciences, Southwestern Medical University, Luzhou 646000, China)*

*(^4^ College of Chemistry and Material Science, Sichuan Normal University, Chengdu 610066, China*)

**Contents**

**Figure. S1** Molecular structure of ornidazole.

**Figure S2.** The total DOSs and PDOSs of ornidazole absorbed on anatase TiO_2_(101) surface under vacuum conditions.

**Figure S3.** The total DOSs and PDOSs of ornidazole absorbed on anatase TiO_2_(001) surface under vacuum conditions.

**Figure S4.** The total DOSs and PDOSs of ornidazole absorbed on anatase TiO_2_(101) surface under neutral conditions.

**Figure S5.** The total DOSs and PDOSs of ornidazole absorbed on anatase TiO_2_(001) surface under neutral conditions.

**Figure S6.** The total DOSs and PDOSs of ornidazole absorbed on anatase TiO_2_(101) surface under acidic conditions.

**Figure S7.** The total DOSs and PDOSs of ornidazole absorbed on anatase TiO_2_(001) surface under acidic conditions**.**

**Figure S8.** The total DOSs and PDOSs of ornidazole absorbed on anatase TiO_2_(101) surface under basic conditions.

**Figure S9.** The total DOSs and PDOSs of ornidazole absorbed on anatase TiO_2_(001) surface under alkaline conditions.

**Table S1.** The net charge Q_1_ of the atom on the imidazole ring and the net charge Q_2_ of the C(2)-N(3) bond atom in the most stable adsorption structure before and after adsorption.

**Figure S1.** Molecular structure of ornidazole.


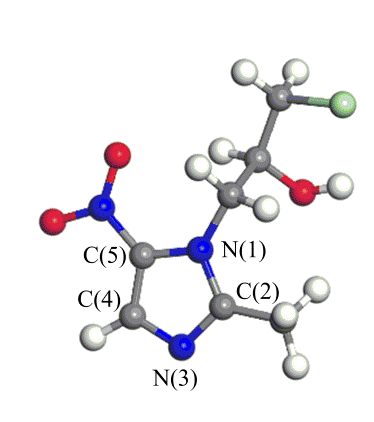


**Figure S2.** The total DOSs and PDOSs of ornidazole absorbed on anatase TiO_2_(101) surface under vacuum conditions.

**
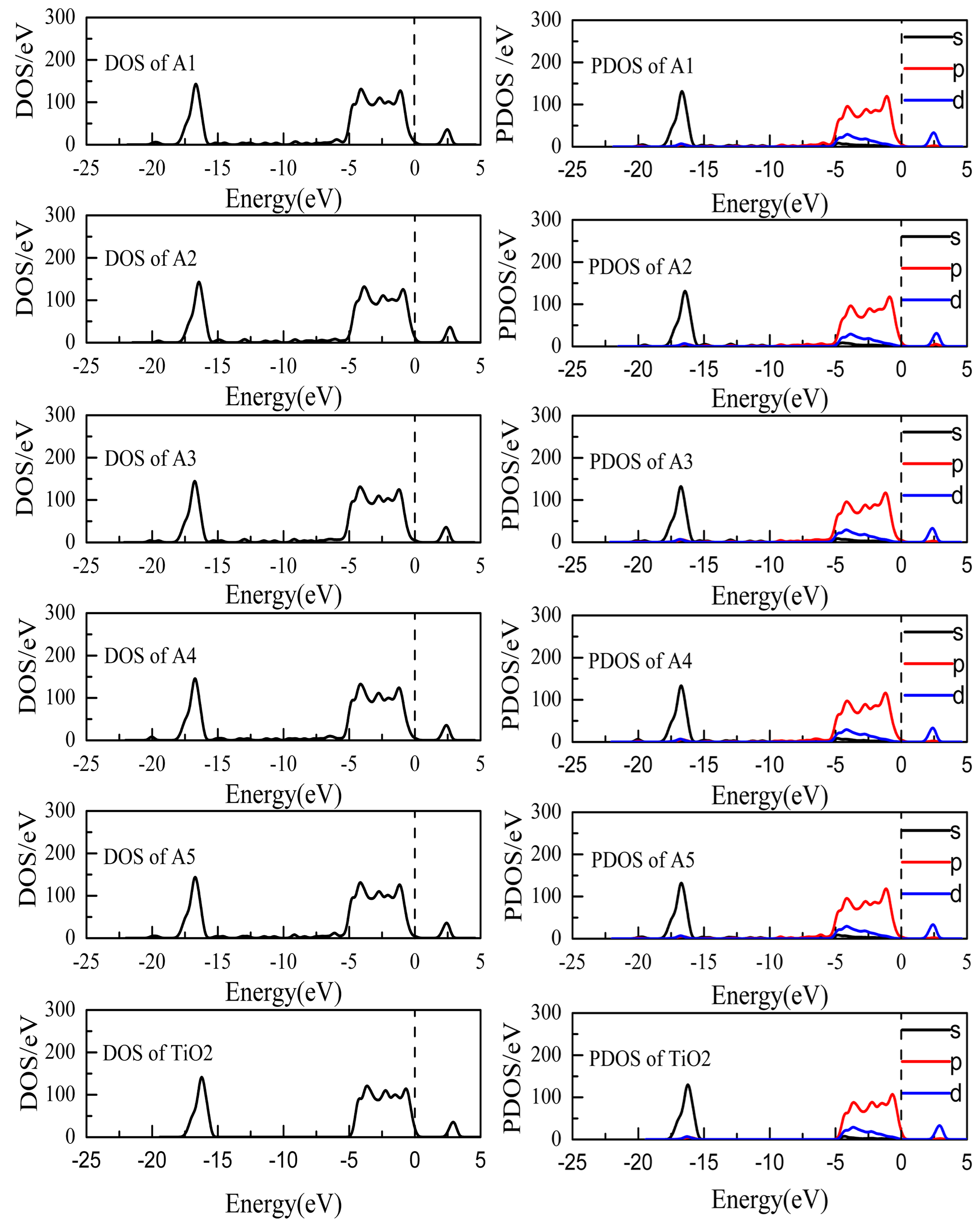
**

**Figure S3.** The total DOSs and PDOSs of ornidazole absorbed on anatase TiO_2_(001) surface under vacuum conditions.


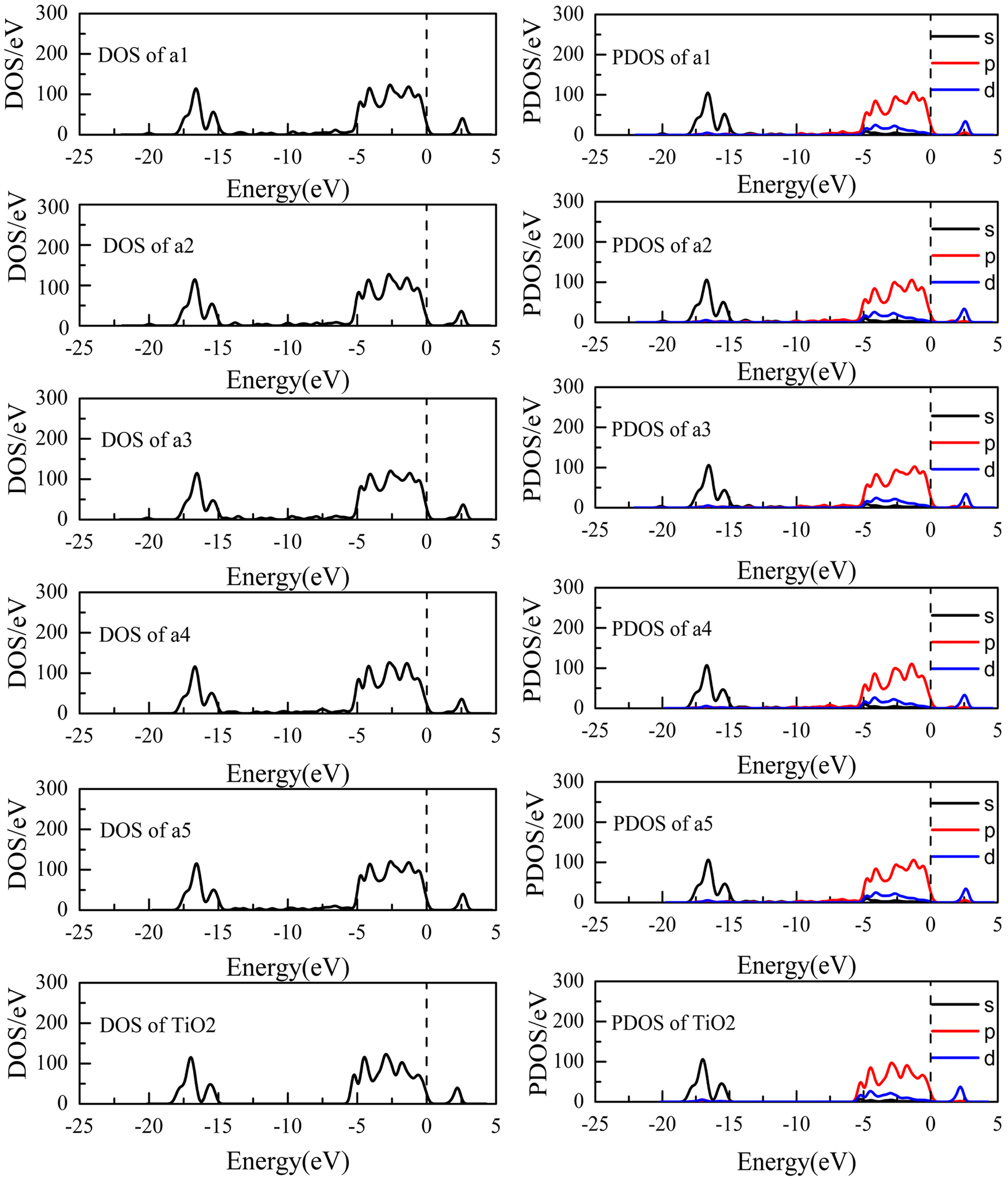


**Figure S4.** The total DOSs and PDOSs of ornidazole absorbed on anatase TiO_2_(101) surface under neutral conditions.

**
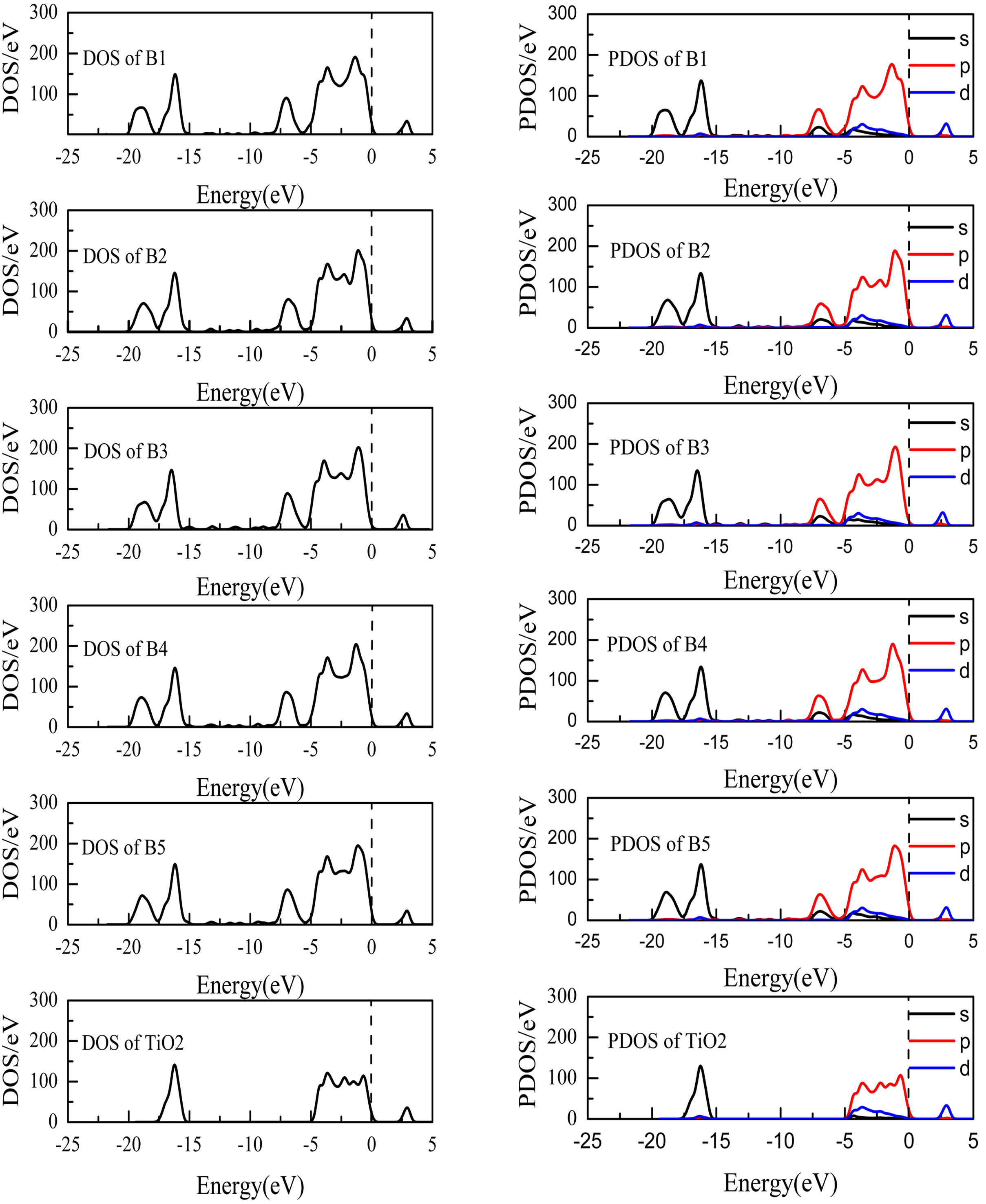
**

**Figure S5.** The total DOSs and PDOSs of ornidazole absorbed on anatase TiO_2_(001) surface under neutral conditions.

**
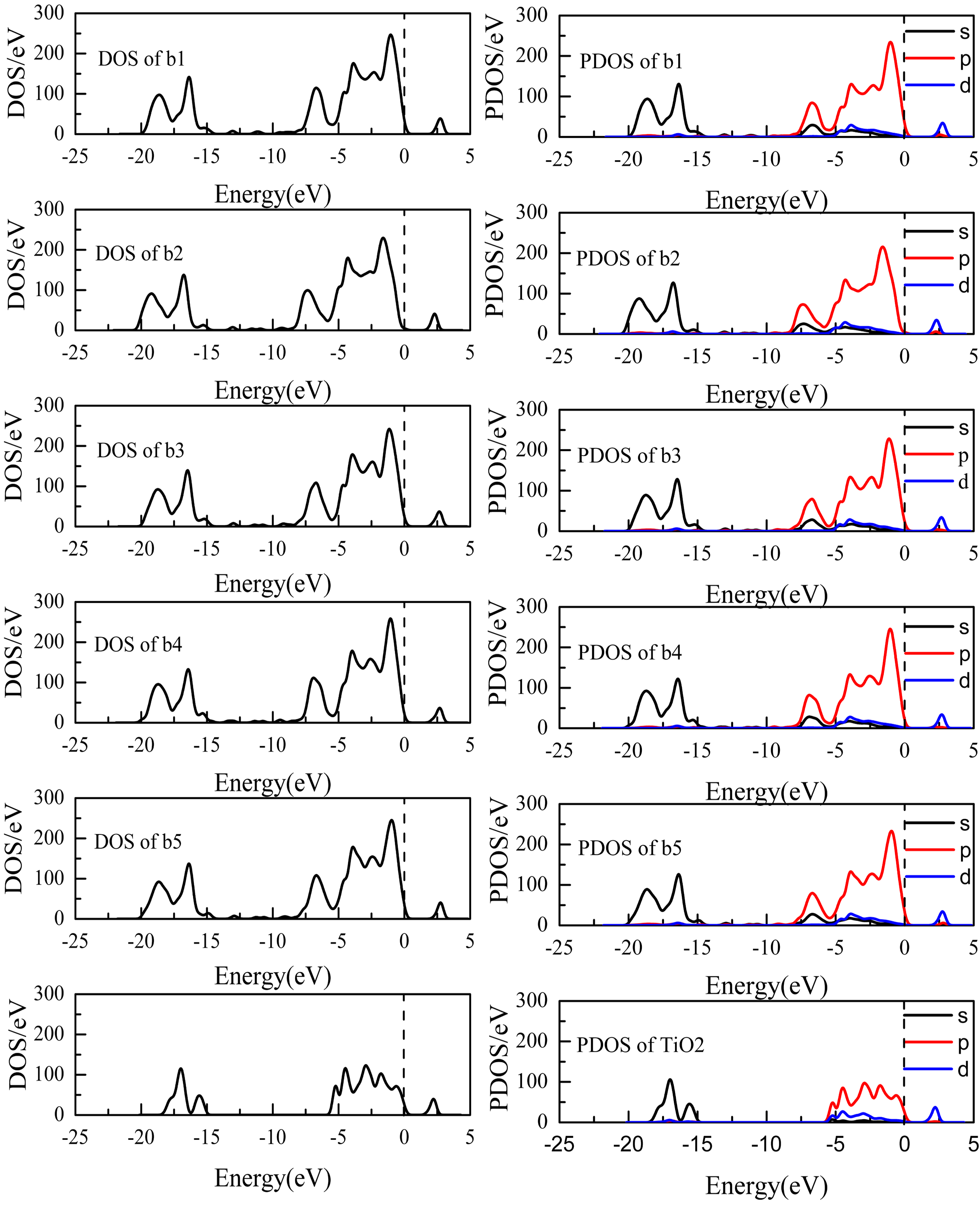
**

**Figure S6.** The total DOSs and PDOSs of ornidazole absorbed on anatase TiO_2_(101) surface under acidic conditions.


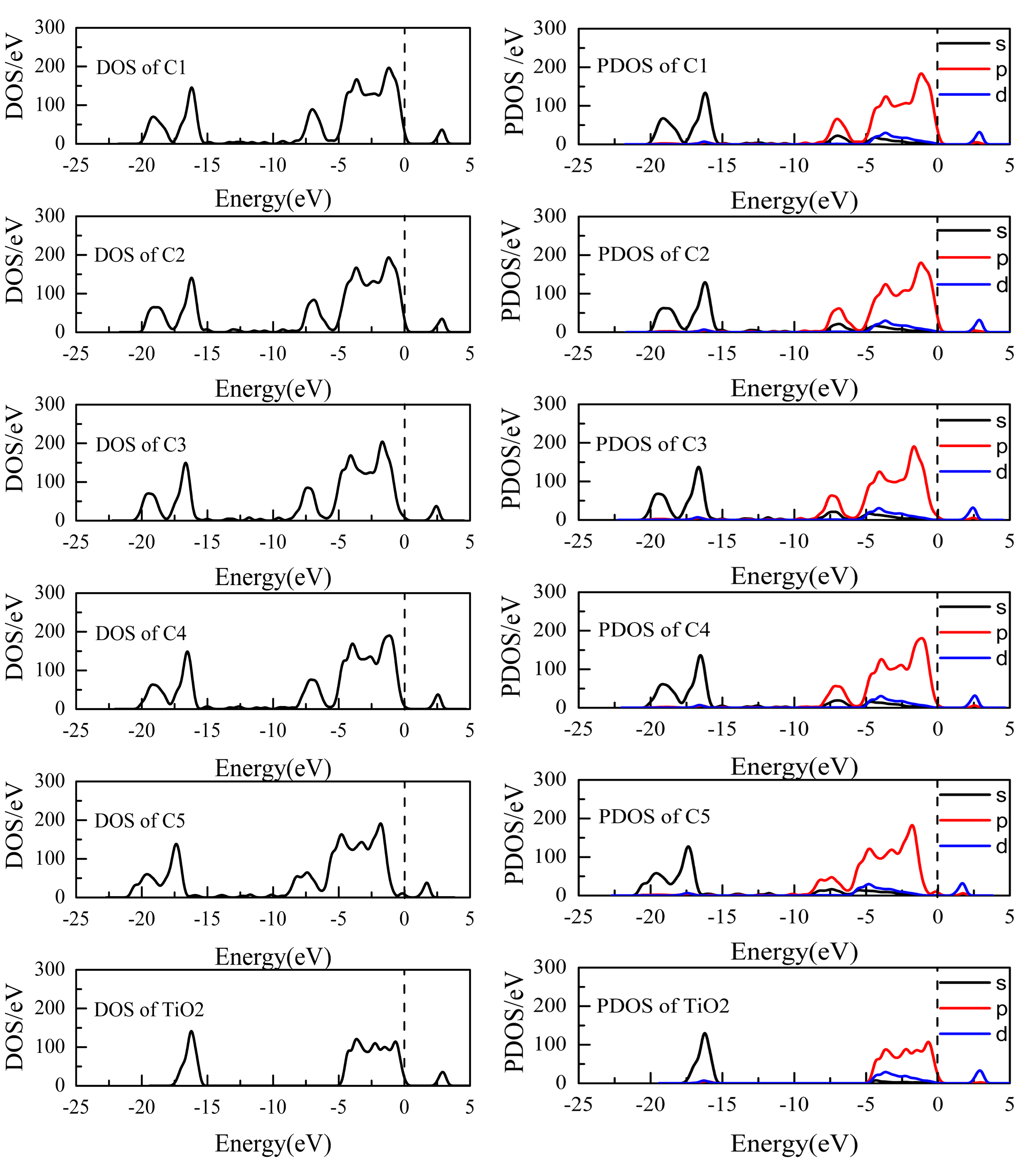


**Figure S7.** The total DOSs and PDOSs of ornidazole absorbed on anatase TiO_2_(001) surface under acidic conditions.

**
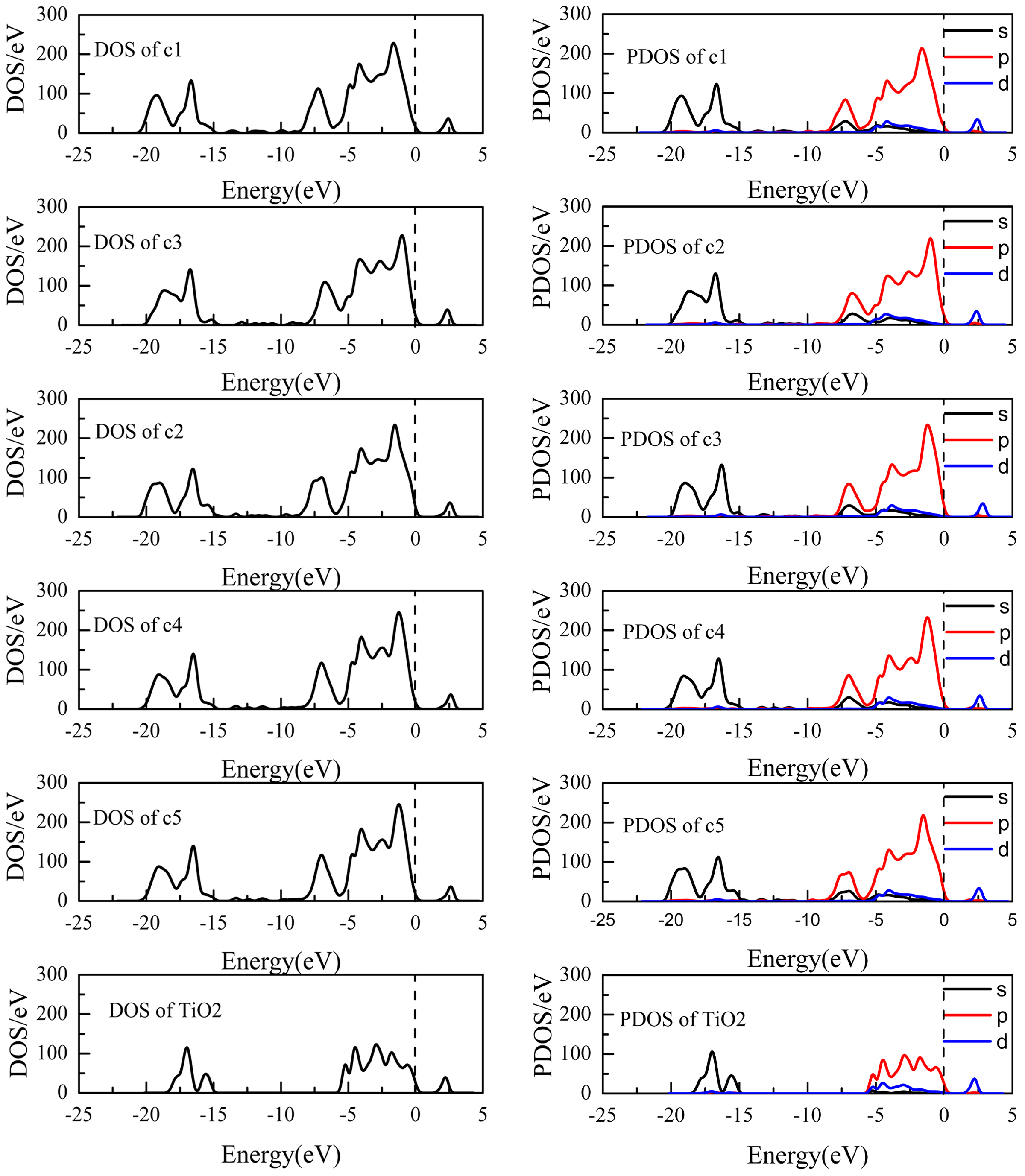
**

**Figure S8.** The total DOSs and PDOSs of ornidazole absorbed on anatase TiO_2_(101) surface under alkaline conditions.

**
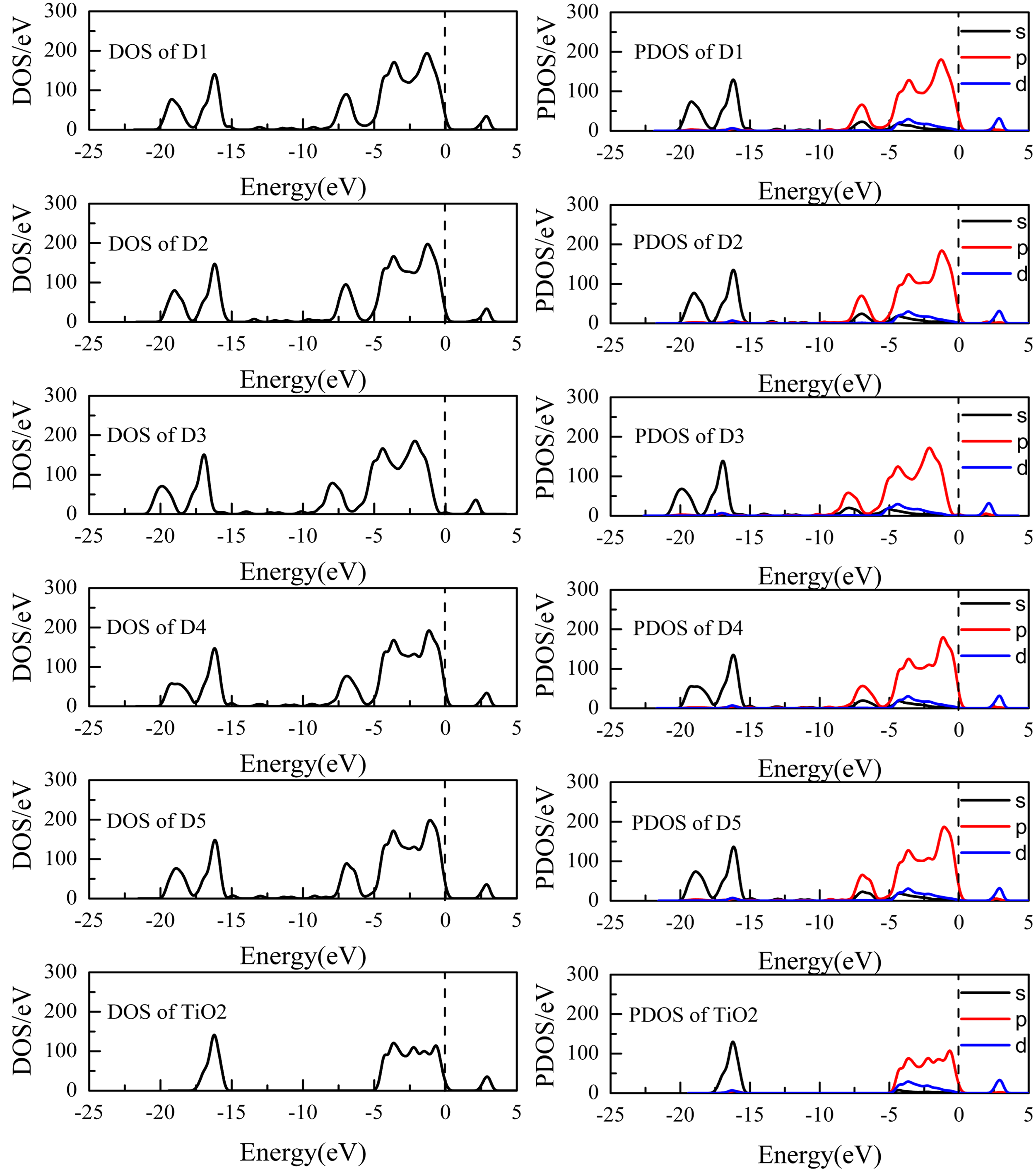
**

**Figure S9.** The total DOSs and PDOSs of ornidazole absorbed on anatase TiO_2_(001) surface under alkaline conditions.


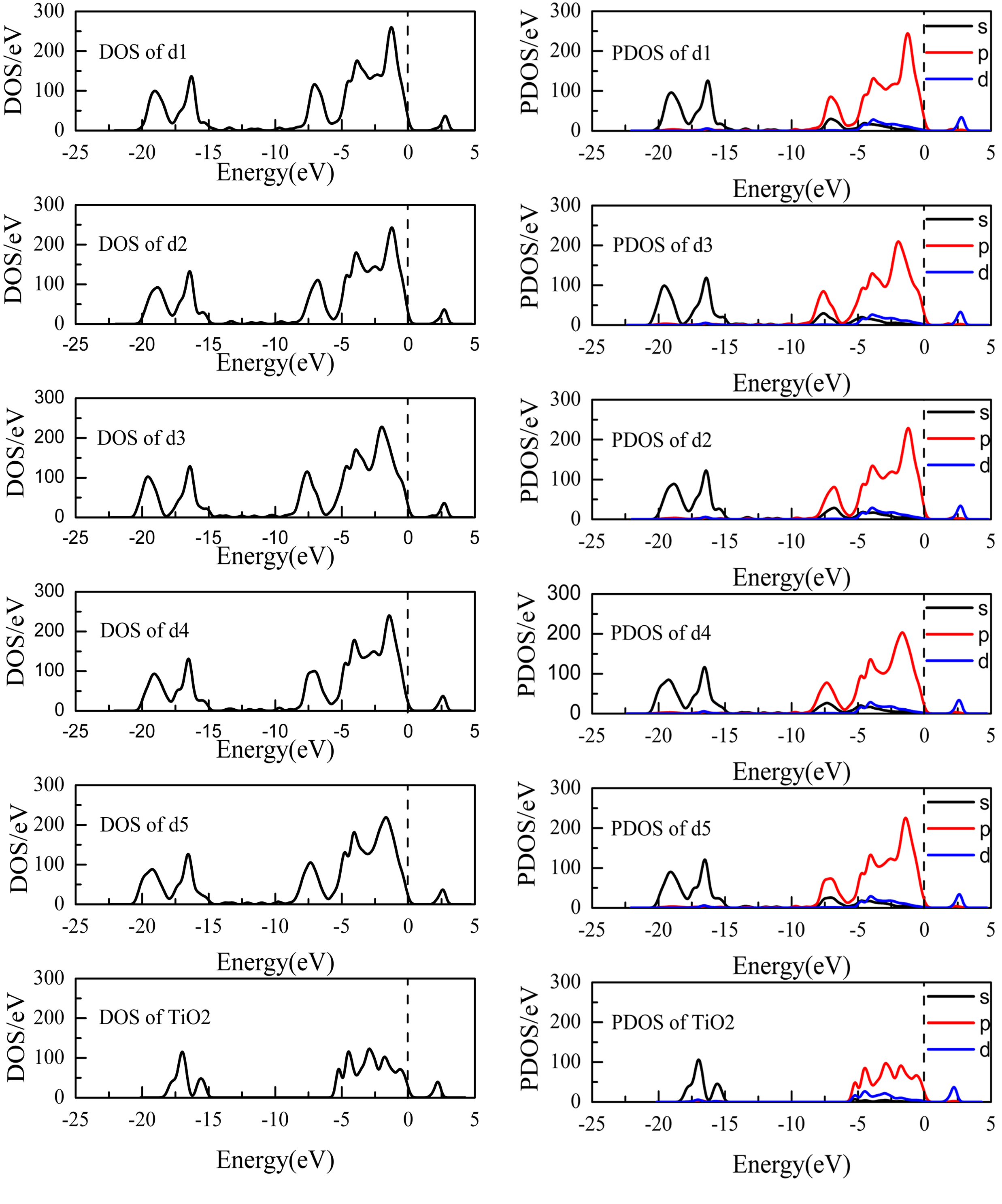


**Table S1.** The net charge Q_1_ of the atom on the imidazole ring and the net charge Q_2_ of the C(2)-N(3) bond atoms before and after adsorption on the most stable adsorption structure.

|  | The net charge before adsorption | The net charge of the stable adsorption configuration under different conditions | | | | | | | |
| --- | --- | --- | --- | --- | --- | --- | --- | --- | --- |
|  |  | A1 | a1 | B5 | b1 | C1 | c2 | D5 | d5 |
| Q_1_ | 0.029 | -0.084 | 0.022 | -0.019 | -0.144 | 0.026 | -0.134 | -0.140 | -0.047 |
| Q_2_ | 0.087 | 0.067 | 0.005 | -0.039 | -0.127 | 0.007 | 0.002 | 0.039 | 0.054 |
